# An improved high-quality genome assembly and annotation of Tibetan hulless barley

**DOI:** 10.1038/s41597-020-0480-0

**Published:** 2020-05-08

**Authors:** Xingquan Zeng, Tong Xu, Zhihao Ling, Yulin Wang, Xiangfeng Li, Shuqing Xu, Qijun Xu, Sang Zha, Wangmu Qimei, Yuzhen Basang, Jiabu Dunzhu, Mingzhai Yu, Hongjun Yuan, Tashi Nyima

**Affiliations:** 1 State Key Laboratory of Hulless Barley and Yak Germplasm Resources and Genetic Improvement, Lhasa, Tibet 850002 China; 2 Research Institute of Agriculture, Tibet Academy of Agricultural and Animal Husbandry Sciences, Lhasa, Tibet 850002 China; 3 Tibet Academy of Agricultural and Animal Husbandry Sciences, Lhasa, Tibet 850002 China; 4 Chengdu Life Baseline Technology Co., LTD, Chengdu, 610041 China; 5 Institute for Evolution and Biodiversity, University of Münster, Münster, 48149 Germany

**Keywords:** Plant genetics, Next-generation sequencing

## Abstract

Hulless barley (*Hordeum vulgare* L. var. *nudum*) is a barley variety that has loose husk cover of the caryopses. Because of the ease in processing and edibility, hulless barley has been locally cultivated and used as human food. For example, in Tibetan Plateau, hulless barley is the staple food for human and essential livestock feed. Although the draft genome of hulless barley has been sequenced, the assembly remains fragmented. Here, we reported an improved high-quality assembly and annotation of the Tibetan hulless barley genome using more than 67X PacBio long-reads. The N50 contig length of the new assembly is at least more than 19 times larger than other available barley assemblies. The new genome assembly also showed high gene completeness and high collinearity of genome synteny with the previously reported barley genome. The new genome assembly and annotation will not only remove major hurdles in genetic analysis and breeding of hulless barley, but will also serve as a key resource for studying barley genomics and genetics.

## Abstract (summary)

**Table:** 

Measurement(s)	RNA • transcriptome • DNA • genome • sequence_assembly • sequence feature annotation
Technology Type(s)	RNA sequencing • DNA sequencing • sequence assembly process • sequence annotation
Sample Characteristic - Organism	Hordeum vulgare

Machine-accessible metadata file describing the reported data: 10.6084/m9.figshare.12162147

## Background & Summary

**Fig. 1:**
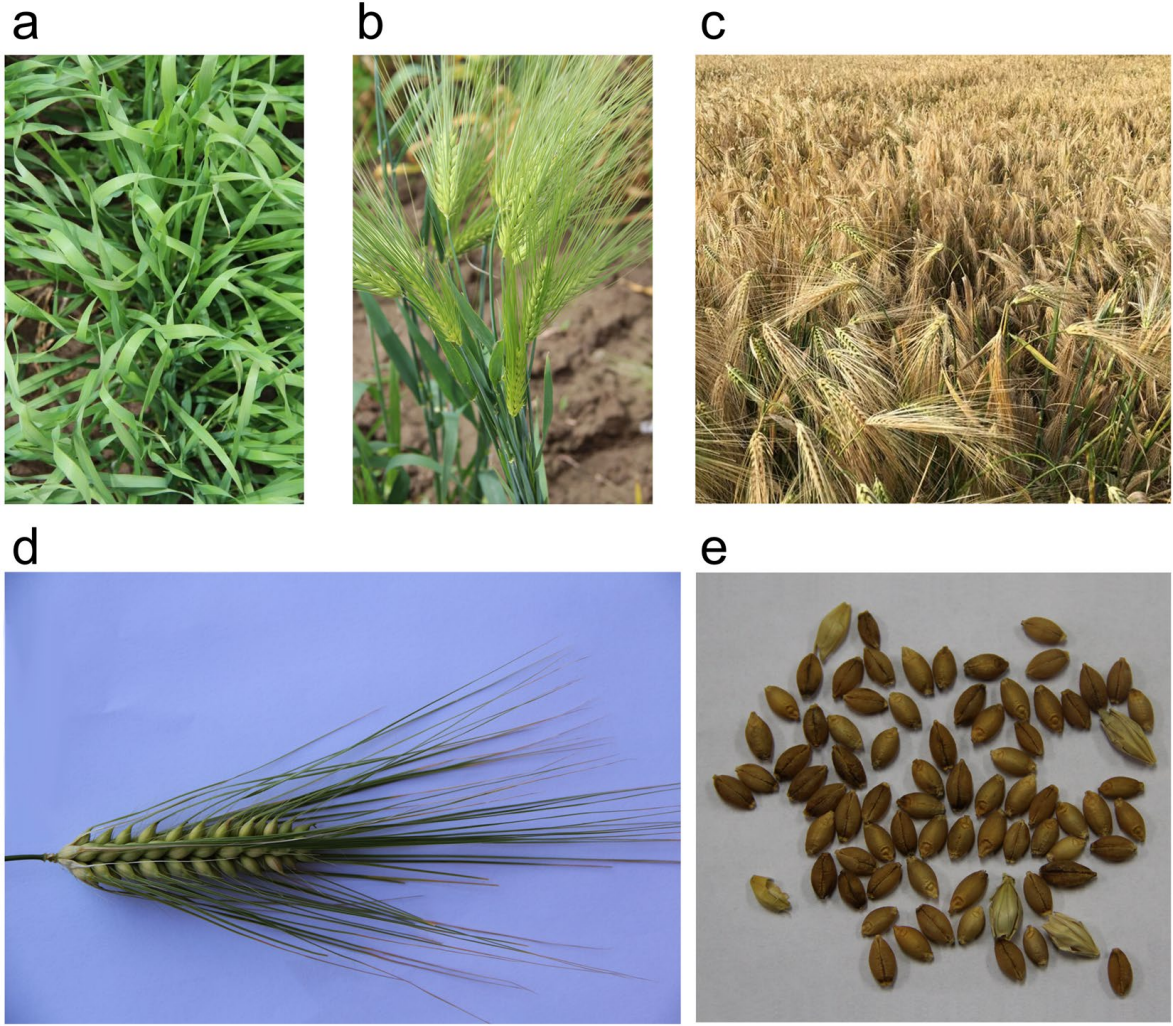
Morphology of a Tibetan hulless barley. These pictures show (**a**) seedling; (**b**) heading stage; (**c**) mature stage; (**d**) filling stage spike; (**e**) grain of a hulless barley cultivated in Tibet.

Hulless barley (*Hordeum vulgare* L. var. *nudum*) is a monophyletically originated variety of barley that has loose husk cover of the caryopses^1^ (Fig. 1). While many domesticated barley varieties are hulled and are mainly used for brewing malt and animal feed, hulless barley has been cultivated on a small scale and used as human food because of the ease in processing and edibility^1^. Although the cultivation of hulless barley is widely distributed, the frequency decreases from east to west^2^. The most frequently cultivated area is the Tibetan plateau, e.g. Nepal and Tibet, where hulless barley accounts for more than 95% of domesticated barley and is the staple food for people and an important livestock feed. Recently, hulless barley is also increasingly attracting attention as a potential crop for the development of value-added products and multiple food applications^3^.

Obtaining genomic sequences is critical for efficient molecular breeding and understanding of the evolutionary history of crops. Recently, studies, including one from our own group, have made significant progress in sequencing the genomes of hulless barley. Using the short-read sequencing approach, the genomes of two hulless barley strains that were grown in Tibet were sequenced and assembled^4,5^. The results suggest that many stress-related genes, which were expanded in hulless barley, might have facilitated the adaptation to the high-altitude environment^4^ and may provide a useful genetic resource for improving barley. Furthermore, by sequencing a population of 437 accessions, a study also showed that the current Tibetan hulless barley cultivars were derived from eastern domesticated barley and were introduced to southern Tibet between 4,500 and 3,500 years ago^6^. However, due to its large genome size and rich in transposable element sequences (80.8–84%)^4,7,8^, the genome assemblies of hulless barley using short-read sequencing approach remain incomplete and fragmented. This constrains the molecular breeding in hulless barley and the use of hulless barley for food applications.

Here, using a long-read sequencing technique (Pacific Biosciences), we sequenced a Tibetan hulless barley cultivar (Lasa Goumang) that has been previously sequenced using short-read, in high coverage (>67X). Using both Pacbio long reads and available Illumina reads, we assembled a significantly improved genome, of which the N50 contig size reached ~1.56 Mb (Table 1). Based on this improved assembly, we re-annotated the protein-coding genes in hulless barley and anchored the scaffolds to a linkage map of the barley cv. Morex^9^. The improved genome assembly and annotations will not only serve as a key resource for exploring the economic and genetic values of hulless barley varieties, but will also advance researches in barley genomics and genetics.

**Table 1:** Titetan hulless barley genome size estimation and assembly statistics in previous study.

	H. vulgare L. var. nudum
Estimated genome size	4.48 Gb
Total size of assembled scaffolds, >200 bp	3.89 Gb
Total sequence length anchored to chromosomes	3.48 Gb
Percent of chromosomal sequences	89.41%
N50 length, scaffolds	242Kb
Longest scaffold	3.07 Mb
Total size of assembled contigs	3.64 Gb
Longest contig	276.95Kb
N50 length, contig	18.07Kb
GC content	44.00%
Repeat content	81.39%
Number of gene models	36,151

## Methods

### DNA isolation, libraries construction and sequencing

The seedlings were germinated from seeds of a Tibetan hulless barley (cultivar Lasa Goumang, NCBI BioSample ID SAMN09914874). Tissues were flash-frozen in liquid nitrogen and stored in the freezer until DNA extraction. DNA was extracted using the cetyltrimethyl ammonium bromide (CTAB) method^10^. The quality of the extracted genomic DNA was checked using electrophoresis on 1% agarose gel and the concentration was quantified using a Qubit fluorometer (Invitrogen, Carlsbad, CA, USA).

Single-molecule real-time (SMRT) long-reads sequencing was performed at NextOmics Technology Corporation (Wuhan, China) with a PacBio sequel sequencer (Pacific Biosciences, Menlo Park, CA, USA). The SMRT Bell library was prepared using a DNA Template Prep Kit (1.0). In total, six 20-kb SMRT Bell libraries were constructed. Genomic DNA (~10 μg) was mechanically sheared to fragments of approximately 20 kb using a Covaris g-TUBE. The fragment size distribution was assessed using a bioanalyzer 2100 12 K DNA Chip assay. A blunt-end ligation reaction followed by exonuclease treatment was conducted to generate the SMRT Bell template. The size-selection of SMRT Bell templates was performed using a BluePippin size-selection system (Sage Science) to enrich large fragments (>10 kb). The quality and quantity of size-selected libraries were assessed on a bioanalyzer12Kb DNA Chip (Agilent) and a Qubit fluorometer (Life Technologies), respectively. The SMRT bell libraries were prepared using the binding kit 2.0 (PacBio p/n 100-862-200) according to the manufacturer’s instructions. The libraries were sequenced using a PacBio Sequel instrument on PacBio SMRT cells v2.0 (Pacific Biosciences, acquiring one movie of 360 min per SMRT cell). The MagBead loading (Pacific Biosciences) method was used to improve the enrichment of the larger fragments. In total, ~300 Gb subreads sequences (average length: 9,358 bp) were generated on 64 SMRT cells.

### RNA isolation and Iso-Seq sequencing

For RNA samples, plants were grown in a climate chamber in the laboratory (Lhasa). Roots, stems and leaves were sampled seven weeks after germination. To have sufficient materials for each RNA sample, we pooled plant tissue from 10 plants. Samples were placed on dry ice during sample collection and stored in −80 °C freezer until RNA isolation. In total, five pooled samples (one root, two stem and two leaf samples) were collected. Samples were ground with liquid nitrogen and total RNA was extracted using TRIzol reagent (Invitrogen) according to manufacturer’s protocol. RQ1 DNase (Promega) was used to remove DNA.

cDNA libraries were prepared using the ClontechSMARTer^®^ cDNA synthesis kit according to the manufacturer’s recommendations. One μg total RNA was used for each of the five samples. Barcoded oligo dT was used to barcode samples. The cDNA products were purified with AMPure PB beads and quality control (QC) was performed on BioAnalyzer 2100 (Agilent). The purified cDNA libraries were pooled in an equal molar ratio. The pooled cDNA (~3.8 μg) was size fractionated using the Sage ELF system. Subsequent re-amplification was performed to yield four libraries (size of 1–2, 2–3, 3–6 and 5–10Kb) to minimize artifacts during large-scale amplification. The pooled PCR products were purified using AMPure PB beads. One to five μg of purified amplicons were subjected to Iso-Seq SMRT Bell library preparation (https://pacbio.secure.force.com/SamplePrep). A total of 17 SMRT cells were sequenced on the PacBio RS II platform using P6-C4 chemistry with 3–4 h movies. In total, 19.68 Gb sequence data (~1.5 million reads) were obtained (Table 2). The average subreads length was 4.0 kb and the average subreads quality was 0.9.

**Table 2:** Iso-Seq library information and sequencing results.

Sample	SRA Accession Number	Platform	Library type	Tissue	Insert size	Polymerase bases (Gb)	Subreads bases (Gb)
m170209_043156_42199	SRR9112621	PacBio Sequel II	SMRT Bell	root, stem and leaf mixed	1–2 kb	1.01	0.94
m170209_085125_42199	SRR9112620			root, stem and leaf mixed	1–2 kb	1.23	1.15
m170210_073652_42199	SRR9112625			root, stem and leaf mixed	1–2 kb	1.31	1.23
m170210_115552_42199	SRR9112624			root, stem and leaf mixed	1–2 kb	1.41	1.32
m170209_131022_42199	SRR9112619			root, stem and leaf mixed	2–3 kb	1.57	1.48
m170209_173334_42199	SRR9112618			root, stem and leaf mixed	2–3 kb	1.48	1.4
m170210_161505_42199	SRR9112623			root, stem and leaf mixed	2–3 kb	1.40	1.32
m170210_203418_42199	SRR9112622			root, stem and leaf mixed	2–3 kb	1.26	1.19
m170211_005331_42199	SRR9112627			root, stem and leaf mixed	3–6 kb	1.59	1.5
m170211_051244_42199	SRR9112626			root, stem and leaf mixed	3–6 kb	1.60	1.52
m170214_153228_42199	SRR9112612			root, stem and leaf mixed	3–6 kb	0.36	0.34
m170214_195529_42199	SRR9112611			root, stem and leaf mixed	3–6 kb	0.36	0.34
m170216_130225_42199	SRR9112614			root, stem and leaf mixed	3–6 kb	1.39	1.31
m170228_215450_42199	SRR9112613			root, stem and leaf mixed	5–10 kb	1.43	1.36
m170301_021333_42199	SRR9112616			root, stem and leaf mixed	5–10 kb	1.12	1.06
m170304_235542_42199	SRR9112615			root, stem and leaf mixed	5–10 kb	1.25	1.19
m170305_041614_42199	SRR9112617			root, stem and leaf mixed	5–10 kb	1.10	1.04
**Total**						20.87	19.67

### Genome assembly

In our assembly workflow, raw bam files from PacBio Sequel were first converted into subreads in fasta format using the PacBio software BAM2fastx. Then we used the falcon package (https://github.com/PacificBiosciences/falcon) to construct the primary assembly. Error correction was performed using an overlap-based strategy and the error-corrected reads were used to construct the contigs (parameters: length_cutoff = 5000; length_cutoff_pr = 10000). To correct errors in the primary assembly, we used the arrow pipeline from the SMRT link 4 toolkit to polish the genome (https://www.pacb.com/products-and-services/analytical-software/smrt-analysis/). The PacBio reads were aligned to the primary assembly using pbalign and variantCaller was used to call variants.

SSPACE^11^ was used to construct scaffolds from contigs. First, we aligned previously sequenced Illumina mate-pair libraries (20 kb and 40Kb fragment long)^4^ to the assembled contigs using bowtie v1.1.2^12^ and constructed scaffolds with SSPACE-STANDARD-3.0. Second, we used PacBio long-reads to further improve the scaffolding using SSPACE-LongRead^13^. After scaffolding, the assembly contains 1,856 scaffolds, with a N50 contig size of 1.56 Mb and an N50 scaffold size of 4.0 Mb. The assembled genome size is 4.0 Gb (Table 3).

**Table 3:** Comparison of the new genome with previously published assemblies of the Tibetan hulless barley genome.

	This study	Zeng *et al*.^4^	Dai *et al*.^5^	cv. Morex^8^
Sequenced genome size (Gb)	4.00	3.89	3.73	4.58
Contig N50 (Kb)	1,563.00	18.07	5.94	79.0
Scaffold N50 (Kb)	4,006.00	242.00	171.1	1,900.00
Repeat proportion (%)	87.48	81.39	NA	80.80
Annotated protein-coding genes	40,457^*^	36,151	46,787	39,734^*^

*Only refer to high confident protein-coding genes.

While the genome size of barley cv. Morex is ~5.1 Gb^9^, our previous work has suggested that the genome size of Tibetan hulless barley is ~4.5 Gb using k-mer analysis^4^ (Table 1). However, it is well-known that genome size estimation from both k-mer approach and flow cytometry can have substantial standard deviations (e.g., 10%)^14,15^. To draw concrete conclusion on genome size differences between hulless barley and cv. Morex, additional experiments are required. However, this is beyond the scope of this study.

**Fig. 2:**
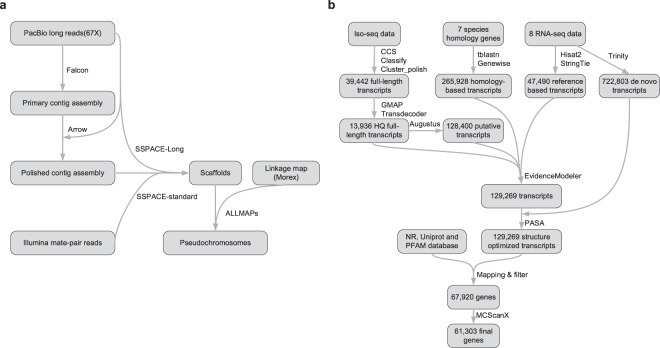
The workflows of genome assembly and annotation used in this study. (**a**) Genome assembly pipeline; (**b**) Protein-coding gene annotation pipeline. Software and tools were indicated at lines, data and database information were shown in rectangles.

**Fig. 3:**
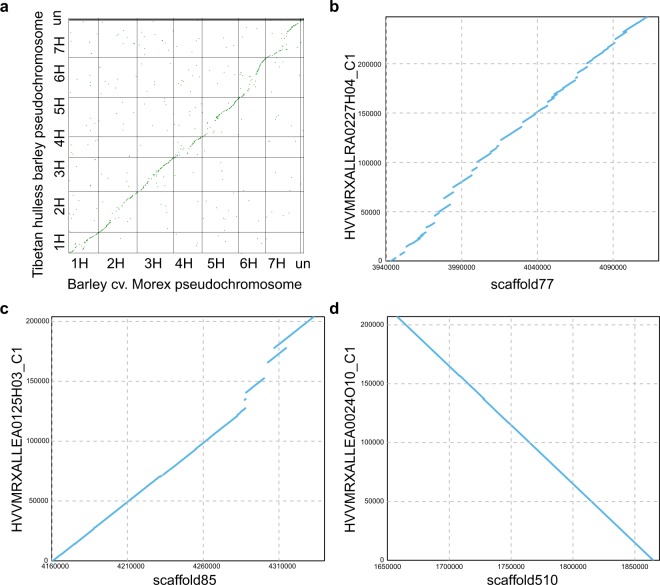
Genome comparison between Tibetan hulless barley and barley cv. Morex. (**a**) The plot shows the LAST alignments of predicted protein-coding genes in barley cv. Morex assembly and Tibetan hulless barley assembly. (**b**–**d**) Dot plots show the MUMMER alignments of Tibetan hulless barley scaffolds and assembled barley cv. Morex bacterial artificial chromosome sequences. Different coverages were shown in B (65%), C (85%) and D (99.9%). The differences can be due to either true sequence divergences or assembly errors.

We further generated pseudochromosomes using the assembled scaffolds and linkage map of barley cv. Morex^9^. We used blastn to map the marker sequences of cv. Morex genome to the scaffolds. Only uniquely mapped markers with coverage greater than 0.8 and identity greater than 0.95 were considered. To anchor the scaffolds to pseudochromosomes, ALLMAPS^16^ was used (Fig. 2a). The synteny comparison between the newly assembled Tibetan hulless barley and barley cv. Morex (Fig. 3a) was performed using CoGe platform^17^ (https://genomevolution.org/coge/).

### Repetitive sequences annotation

Repetitive DNA sequences are highly abundant in many organisms and their variations in abundancy resulted in remarkable genome size variations in plant^18^. In many Gramineae crop plants, repetitive elements represent more than 80% of their genome^8,19,20^. Repetitive elements can be classified as simple repeats and transposable elements (TE). Using tandem repeats finder^21^, we annotated ~155 Mb (3.89%) sequences as simple repeats. To annotate TE, we used both homology-based and *de novo* TE annotation tools: RepeatMasker^22^, RepeatProteinMask^22^, RepeatModeler (http://www.repeatmasker.org/RepeatModeler/) and LTR_FINDER^23^. For RepeatMasker, Repbase 21.01^24^ was used. In total, ~87.5% of the assembled genome were identified as TE (Table 4).

**Table 4:** Repetitive element annotation statistics.

TE class	TE order	TE family	cv. Morex	Tibetan hulless barley (this study)
Class I: retrotransposon	LTR retrotransposon	Copia	22.3	23.3
		Gypsy	44.2	46.6
		unclassified LTR	0.2	0.3
	non-LTR retrotransposon	LINE	1.3	1.3
		SINE	0	0
	Sum		**68**	**71.5**
Class II: DNA Transposon	DNA Transposon super-families		6.5	6.1
	Helitron		0.03	0.04
	other DNA transposon		1.6	1.7
	Sum		**7.1**	**7.8**
Unclassified			8	8.1
Total			**83.1**	**87.5**

### Protein-coding gene prediction

For the annotation of protein-coding genes, we used a previously established gene annotation pipeline (Fig. 2b) with minor modifications^4^. For *de novo* gene prediction, we first extracted the full-length transcripts from the Iso-Seq data using SMRTLINK. In total, 39,442 full-length transcripts were obtained and were subsequently aligned to the assembled genome using GMAP^25^. Among the 38,013 aligned transcripts, we removed all transcripts that had coverage less than 0.9 or sequence identity less than 0.85. This resulted in 14,099 high-quality full-length transcripts, which were used for open reading frame (ORF) prediction by TransDecoder (https://github.com/TransDecoder). In total, 13,936 (98.8%) transcripts contained at least one open reading frame (ORF) that is larger than 50 amino acids. These ORF containing transcripts were then assigned to 9,360 genes, which were considered as authentic genes. The authentic genes were then used for training the gene prediction models using AUGUSTUS v3.2.3^26^. Based on the trained models, AUGUSTUS predicted 128,400 putative genes.

For homology based gene prediction, we used the protein sequences of seven monocot species (*Triticum urartu* (progenitor of wheat A genome)^20^*, Triticum tauscii* (progenitor of wheat D genome)^27^, *Brachy podiumdistachyon*^28^, *Hordeum vulgare*8, *Oryza sativa*^29^, *Sorghum bicolor*^30^ and *Zea mays*^31^) from public databases. All protein sequences were aligned to the hulless barley genome using tblastn^32^. The gene structure was predicted using GeneWise^33^ with the input protein sequence as reference.

To provide further evidence for evaluating the predicted gene models, we assembled the transcriptome using available RNA-seq Illumina short-reads from different libraries^34^. The transcriptome was assembled using both reference-guided approach (mapping: hisat2^35^, assembly: stringtie^36,37^) and *de novo* approach (Trinity pipeline^38^). The reference-guided approach resulted in 47,490 transcripts and the *de novo* approach resulted in 722,803 transcripts.

The full-length transcripts from Iso-Seq, assembled transcripts from short-reads were used as evidence to evaluate the predicted gene models using EVidenceModeler^39^. For the data integration, evidence from different sources was assigned to different weight parameters: 20 for Iso-Seq assembly, 8 for short-reads assembly, 5 for homology-based prediction, 2 for AUGUSTUS gene prediction. In total, 129,269 transcripts were obtained, and the structural optimization was performed using PASA. We removed transcripts that either do not show any homology to sequences in nr or uniport database (blast results with identity > = 50% and coverage > = 50%) or have no protein sequences containing any Pfam^40^ domain (hmmer results with e-value < = 1e-5). For each gene, only the transcript with the longest protein sequences was kept. The tandem duplicated genes were identified using MCScanX. Genes (4,530) that contain large TE sequences (90% coverage) were discarded. The pipeline generated 61,303 genes.

We further classified these genes into high-confidence (HC) genes (40,457), which are likely true protein-coding genes, and less reliable low-confidence (LC) genes (20,846), which potentially are fragmented genes, pseudogenes and/or non-coding genes. This was done in a two-step procedure as described previously^8^.

We annotated putative functions of the 61,303 protein sequences using public databases, including nr, KEGG^41^, SwissProt^42^, Trembl^42^, GO^43^, PFAM^40^, and InterPro^44^. Blastp was used to compare the predicted protein sequences with the protein databases(e-value < = 1e-5). Blast2GO^45^ was used to annotate the GO terms using nr database (downloaded in December 2017) with default parameters. The protein domains were annotated using PfamScan^46^ and InterProScan^47^ based on InterPro protein databases, including CATHGene3D^48^, HAMAP^49^, PANTHER^50^, PIRSF^51^, PRINTS^52^, ProDom^53^, PROSITE^54^, SMART^55^, SUPERFAMILY^56^ and TIGRFAMs^57^.

### Non-coding gene prediction

tRNAs were annotated using tRNAscan-SE v1.3.1^58^ and rRNAs were annotated using blastn with the rRNA sequences from *Arabidopsis thaliana* and *Oryza sativa* (5S rRNA: AJ307354, 5.8S rRNA: AJ232900, 18S rRNA: X16077, 28S rRNA: AH001750). In addition, we also used INFERNAL to predict the miRNA and snRNA.

## Data Records

The genomic Pacbio sequencing data (SRS3725794) and Iso-Seq sequencing data (SRS4809149) are available in NCBI Sequence Read Archive under SRP159129^59^. The available Illumina genome sequencing data that was deposited under SRP055042^4,60^ was used in our genome assembly and validation processes. The final genome assembly and annotation was deposited at NCBI GenBank under SDOW00000000^61^ and NCBI Assembly under GCA_004114815.1^62^. The previously generated RNA sequencing data (deposited in NCBI’s Sequence Read Archive under SRP074870^63^) was used in our genome annotation steps. All the files in this project, such as the assembled scaffolds, repeat annotation, gene predictions and gene function annotations were uploaded to figshare^64^.

## Technical Validation

We evaluated the quality of the new assembly using three independent approaches. First, we mapped 682.57 Gb previously generated genomic Illumina paired-end reads^4,60^ to the assembled genome. Overall, ~99.9% of paired-end reads were mapped to the genome concordantly. Second, using the mapped short reads, we estimated the quality value (QV) of the assembly using a previously described method^65^ in which erroneous bases in the genome assembly were identified based on the variant calling software Genome Analysis Toolkit (GATK)^66^. The estimated base pair error rate is 6.3E-06, suggesting high accuracy of the assembly at base-pair level. Third, we mapped available RNA-seq Illumina reads to the new assembly^34^ using bowtie2 (parameters:--sensitive --score-min L,0,-0.1 -k 200 --no-discordant --gbar 99999999 --dpad 0 -p 24 --no-mixed -X 1000 --mp 1,1 -I 1 --np 1). The mapping rate with the new assembly (more than 73.6%) is more than 10% higher than the previous assembly. Fourth, we also mapped the PacBio long reads to the new assembly using blasr (parameters: -m 4 --minMatch 8 --minPctIdentity 70 --bestn 1 --nCandidates 20 --maxScore -500). Overall, 91.2% PacBio long-reads can be mapped back to the assembly. Fifth, we downloaded the assembled bacterial artificial chromosome (BAC) sequences from barley cv. Morex^67^ and mapped them back to the new hulless barley assembly using mummer^68^ (Fig. 3b–d). Among 299 BACs that were larger than 200 kb and can be mapped back to barley cv. Morex, 74.6% showed high collinearity with the new hulless barley assembly (coverage greater than 60%). Sixth, we also used BUSCO^69^ to assess the genome completeness. Among 1,440 conserved eukaryotic core genes, 1,378 (95.7%) were complete, 15 were fragmented and 47 were missing in the hulless barley genome assembly. Together, the results suggested that the newly assembled hulless barley genome is of high quality and will serve as a key resource for future research in barley genetics and genomics.

## Data Availability

The software mentioned in methods section are described blow. **1) falcon**: version 1.2.4, parameters: (length_cutoff=5000, length_cutoff_pr=10000); **2) pbalign**: contained in SMRT Link 4 toolkit, parameters:(--algorithm=blasr); **3) variantCaller**: contained in SMRT Link 4 toolkit, parameters:(--algorithm=arrow); **4) bowtie**: verion 1.1.2, default parameters; **5) SSPACE-STANDARD**: version 3.0, parameters:(-p 1 -g 2); **6) SSPACE-LongRead**: version 1-1, default parameters; **7) blasr**: version 1.3.1.121193, default parameters; **8) GATK**: version 4.0.0.0, default parameters; **9) nucmer**: contained in mummer version 4.0.0beta2, default parameters; **10) BUSCO**: version 3, parameters:(-l embryophyta_odb9); **11) Tandem repeats finder**: version 409, default parameters; **12) RepeatMasker**: version open-4.0.7, parameters: (-nolow -no_is -norna -engine ncbi -parallel 1); **13) RepeatProteinMask**: version open-4.0.7, parameters:(-engine ncbi -noLowSimple -pvalue 0.0001); **14) RepeatModeler**: version open-1.0.11, parameters:(-engine ncbi -pa 9); **15) ltr_finder**: version 1.06, parameters:(-w 2); **16) GMAP**: version 2017-05-08, parameters:(-z sense_force -f samse -n 0); **17) TransDecoder**: version 4.0.1, default parameters; **18) augustus**: version 3.2.3, default parameters; **19) tblastn**: version 2.6.0+, parameters:(-evalue 1e-5 -seg no); **20) GeneWise**: version 2.4.1, default parameters; **21) hisat2**: version 2.1.0, parameters:(--dta --no-discordant --no-mixed); **22) StringTie**: version 1.2.4, default parameters; **23) EVidenceModeler**: version 1.1.1, parameters:(--weights:PROTEIN GeneWise:5, TRANSCRIPT StringTie:8, ABINITIO_PREDICTION AUGUSTUS:2, OTHER_PREDICTION transdecoder:20); **24) Trinity**: version 2.4.0, parameters: (--group_pairs_distance 500 --path_reinforcement_distance 80 --min_glue 3 --min_kmer_cov 3 --min_contig_length 100 --KMER_SIZE 25 --bflyHeapSpaceInit 1G --bflyHeapSpaceMax 4G --bfly_opts “-V 5 --edge-thr=0.1 --stderr”); **25) PASA**: version 2.1.0, default parameters; **26) MCScanX**: latest version, default parameters; **27) tRNAscan-SE**: version 1.3.1, default parameters; 28) infernal: version 1.1.1, default parameters. **28) tRNAscan-SE**: version 1.3.1, default parameters; 28) infernal: version 1.1.1, default parameters.
